# NAB2 is a novel immune stimulator of MDA-5 that promotes a strong type I interferon response

**DOI:** 10.18632/oncotarget.23725

**Published:** 2017-12-15

**Authors:** Anne Oberson, Lorenzo Spagnuolo, Viola Puddinu, Winfried Barchet, Karola Rittner, Carole Bourquin

**Affiliations:** ^1^ Chair of Pharmacology, Department of Medicine, Faculty of Science, University of Fribourg, 1700 Fribourg, Switzerland; ^2^ School of Pharmaceutical Sciences, University of Geneva, University of Lausanne, 1211 Geneva, Switzerland; ^3^ Department of Anesthesiology, Pharmacology and Intensive Care, Faculty of Medicine, University of Geneva, 1211 Geneva, Switzerland; ^4^ German Center for Infection Research, Cologne-Bonn, Germany; ^5^ Institute for Clinical Chemistry and Clinical Pharmacology, University of Bonn, Germany; ^6^ Transgene S.A., Parc d’Innovation, CS80166, 67405 Illkirch-Graffenstaden Cedex, France

**Keywords:** poly(I:C), NAB2, pattern-recognition receptors, interferon, cancer immunotherapy

## Abstract

Novel adjuvants are needed to increase the efficacy of vaccine formulations and immune therapies for cancer and chronic infections. In particular, adjuvants that promote a strong type I IFN response are required, since this cytokine is crucial for the development of efficient anti-tumoral and anti-viral immunity. Nucleic acid band 2 (NAB2) is a double-stranded RNA molecule isolated from yeast and identified as an agonist of the pattern-recognition receptors TLR3 and MDA-5. We compared the ability of NAB2 to activate innate immunity with that of poly(I:C), a well-characterized TLR3 and MDA-5 agonist known for the induction of type I IFN. NAB2 promoted stronger IFN-α production and induced a higher activation state of both murine and human innate immune cells compared to poly(I:C). This correlated with a stronger activation of the signalling pathway downstream of MDA-5, and IFN-α induction was dependent on MDA-5. Upon injection, NAB2 induced higher levels of serum IFN-α in mice than poly(I:C). These results suggest that NAB2 has the potential to become an efficient adjuvant for the induction of type-I IFN responses in therapeutic immunization against cancer or infections.

## INTRODUCTION

A major focus of current research is the development of novel and more effective immune stimulants that can be used as adjuvants, in order to increase vaccine efficacy and safety [1]. Moreover, considering the rising importance of immunotherapy for cancer treatment, there is a need for effective immune modulators that can improve current anti-cancer immunotherapeutic approaches. A class of molecules that were shown to be highly efficient adjuvants are the pathogen-associated molecular patterns (PAMPs) [2]. PAMPs comprise molecular structures, such as specific lipids moieties, nucleic acid structures and lipoproteins, that are highly conserved across a wide range of microorganisms, and whose recognition by immune cells can play a critical role in the early detection of invading pathogens. Indeed, PAMPs can trigger a specific group of innate immune sensors, called pattern-recognition receptors (PRRs), which, upon stimulation, promote the initiation of the innate immune response and the development and coordination of the subsequent adaptive immune response [3]. Different types of PRRs exist, depending on their subcellular localization and the target they recognize. Nucleic acid-sensing PRRs are triggered by specific DNA and RNA structures, and are of particular importance for the detection of viral infections [4]. Indeed, a consequence of nucleic acid sensing by immune cells is the production of key anti-viral cytokines such as type I IFNs, mainly by conventional and plasmacytoid dendritic cells (cDCs and pDCs) and macrophages [5]. Importantly, type I IFNs are central linkers of the innate and adaptive immune response, and are known to mediate prominent anti-tumoral effects [6]. Thus, the stimulation of nucleic acid sensors, through synthetic molecules or naturally occurring nucleic acids, is of a special interest for cancer therapy.

Polyriboinosinic–polycytidylic acid (poly(I:C)) is a synthetic double-stranded RNA (dsRNA) that is an agonist for the nucleic acid sensors Toll-like receptor 3 (TLR3) [7] in the endosome and Melanoma Differentiation-Associated protein 5 (MDA-5) [8], a cytosolic receptor belonging to the RIG-I-like receptor (RLR) family. Poly(I:C) is currently the most consistently active variant of polynucleotide products that, signalling through TLR3 and MDA-5, is able to induce a strong IFN activity [9]. Preclinical tests have shown that poly(I:C), included as adjuvant in cancer vaccines [10] but also as a stand-alone treatment [11], is able to promote an efficient anti-tumoral immune response, activating DCs, NKs and T cells [12, 13]. However, the use of poly(I:C) or poly(I:C) analogues in clinical settings is still limited, mainly due to its unfavourable toxicity profile [14, 15]. Furthermore, the high variability of the poly(I:C) effects that is associated with the use of different product batches, as often observed even in preclinical tests, is a critical downside of this PRR agonist.

Recently, a novel TLR3 and MDA-5 agonist was identified and isolated from a microsomal *Saccharomyces cerevisiae* yeast extract. This molecule turned out to be a 4.6-kb dsRNA molecule with a sequence identical to a yeast dsRNA virus belonging to the *Totoviridiae* family, and it was termed nucleic acid band 2 (NAB2) [16]. NAB2 was shown to induce the secretion of pro-inflammatory cytokines and type I IFNs in human monocyte-derived DCs, signalling through TLR3 and MDA-5. Moreover, NAB2 injection augmented the efficacy of a prophylactic anti-cancer vaccine in a transplantable tumor mouse model, increasing tumor rejection rate and survival [16].

The aim of this work was to compare the efficacy of NAB2 and poly(I:C) as innate immune stimulants: we set out to assess whether NAB2 could represent a more reliable adjuvant, able to circumvent the lack of reproducibility typically associated with different poly(I:C) batches and to consistently induce a strong cytokine response. We report here that NAB2, through an enhanced activation of MDA-5 downstream signalling, promoted a stronger IFN-α production by murine and human innate immune cells *in vitro*, and a significant increase in IFN-α serum levels *in vivo*, than poly(I:C). We propose that NAB2 represents a valid alternative to poly(I:C) as nucleic acid-sensor agonist, with a potentially improved efficacy in the immune-based treatments of cancer and as vaccine adjuvant.

## RESULTS

### NAB2 induces stronger type I IFN responses than the same doses of poly(I:C)

**Figure 1:**
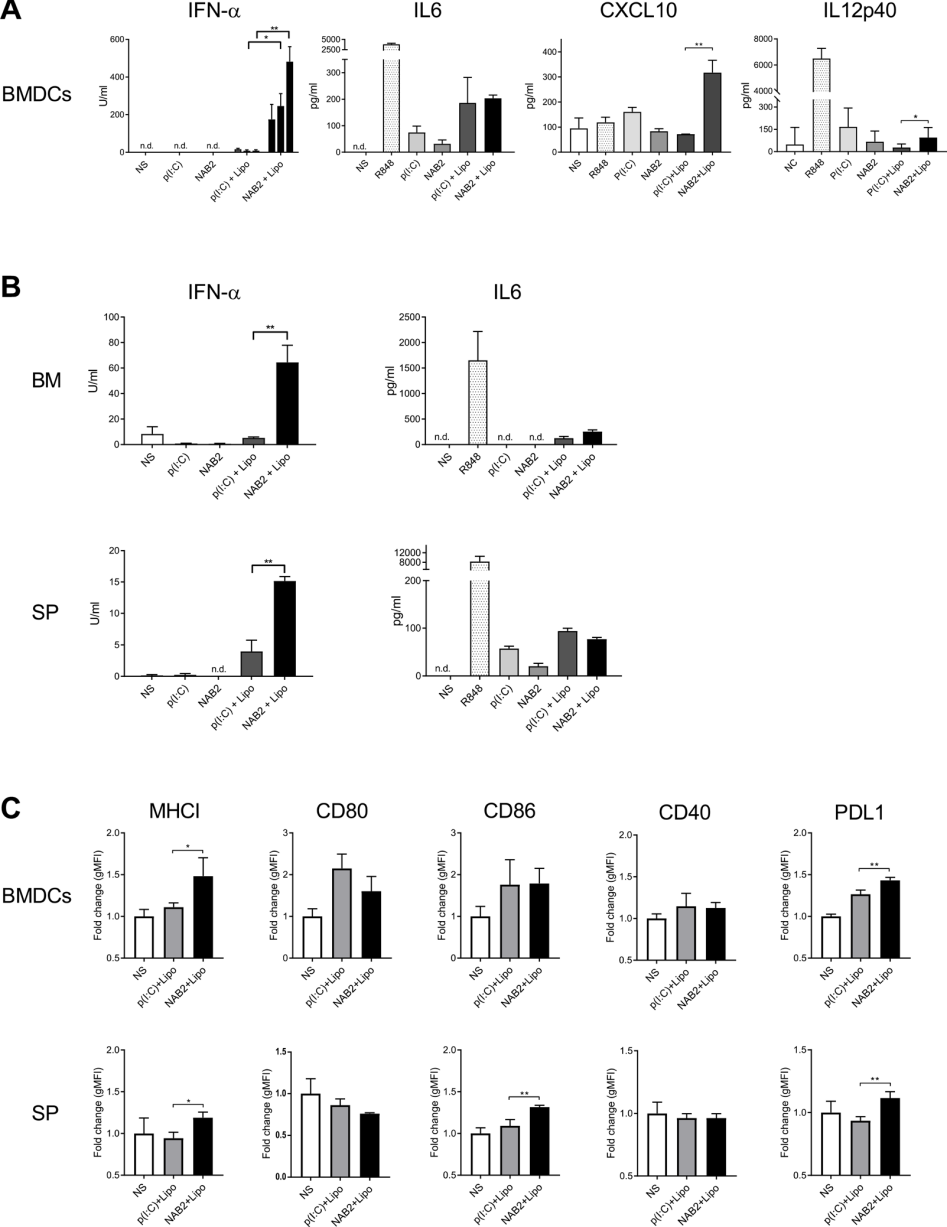
NAB2 enhances type I IFN response in murine immune cells *(***A**) Cytokine levels in the supernatant of BMDC, stimulated for 24 h with 0.5, 2 and 5 µg/ml of NAB2 or poly(I:C), with and without lipofectamine, measured by ELISA. For IL-6, CXCL10 and IL-12p40 plots, results of stimulation with 2 µg/ml NAB2 or poly(I:C) are represented. Mean ± SEM of triplicates are shown. Data are representative of four independent experiments. Significance was measured by two-way ANOVA with Bonferroni post-test correction or Student *t*-test, respectively for IFN-α and for IL-6, CXCL10 and IL-12p40. (**B**) Cytokine levels in the supernatant of freshly isolated bone marrow cells (BM) and splenocytes (SP), stimulated for 24 h with 2 µg/ml of the indicated agonists. Mean ± SEM of triplicates are shown. Data are representative of at least 2 independent experiments. (**C**) Surface expression of activation markers on BMDC (upper panels) and splenic DC (SP) upon 24 h stimulation with 2 µg/ml of the indicated agonists, measured by flow cytometry. Geometric mean fluorescence intensity (gMFI) fold increase compared to unstimulated cells is plotted. Bar plots represent pooled data of at least three independent experiments performed in triplicate. Mean ± SEM are shown. Significance was measured by Student *t*-test. ^*^p < 0.05, ^**^p < 0.01. n.d., cytokine levels below detection limit.

To compare the cytokine-producing ability of poly(I:C) and NAB2, we first assessed the effect of the two agonists on murine bone marrow-derived dendritic cells (BMDC). To monitor RLR and TLR activity upon stimulation, we measured IFN-α and IL-6 production, respectively, because they represent the signature cytokines for each of these pathways [17]. IFN-α production was clearly increased in a dose-dependent manner in cells treated with NAB2 and the transfection reagent lipofectamine, whereas we did not observe any difference in IL-6 production in NAB2 and poly(I:C)-treated cells (Figure 1A). Furthermore, CXCL-10, that is known to be induced by type I and type II IFNs [18], was also increased upon NAB2 stimulation compared to poly(I:C). Without lipofectamine we observed only marginal cytokine production upon both NAB2 and poly(I:C) stimulation. We did not observe a detectable IL-10 production (data not shown), and only a marginal IL-12p40 production (Figure 1A) with NAB2 and poly(I:C). We observed a similar effect using freshly isolated murine bone marrow cells and splenocytes: the stimulation of both these primary cell populations with NAB2 induced a higher IFN-α production and only a marginal amount of IL-6, compared to poly(I:C) (Figure 1B).

To characterize the phenotype of dendritic cells stimulated with NAB2 and poly(I:C), we evaluated the surface expression of different activation markers and co-stimulatory molecules on BMDC (Figure 1C upper panels) and splenic DC (Figure 1C lower panels). In line with the cytokine profile of stimulated BMDC, we observed a higher MHC class I and PD-L1 expression upon NAB2-mediated stimulation compared to poly(I:C) (Figure 1C), both in BMDC and splenic DC, suggesting an enhanced DC maturation. In contrast, NAB2 induced a similar surface expression of the co-stimulatory molecules CD80, CD86 and CD40 in BMDC, and only splenic DC showed an increased CD86 expression upon NAB2 stimulation. In summary, we show that NAB2 stimulation promotes a more pronounced type I IFN response in different types of murine immune cells, and a more marked activation state of BMDC, compared to poly(I:C).

### NAB2-activated BMDC induce antigen-specific T-cell production of effector cytokines

**Figure 2:**
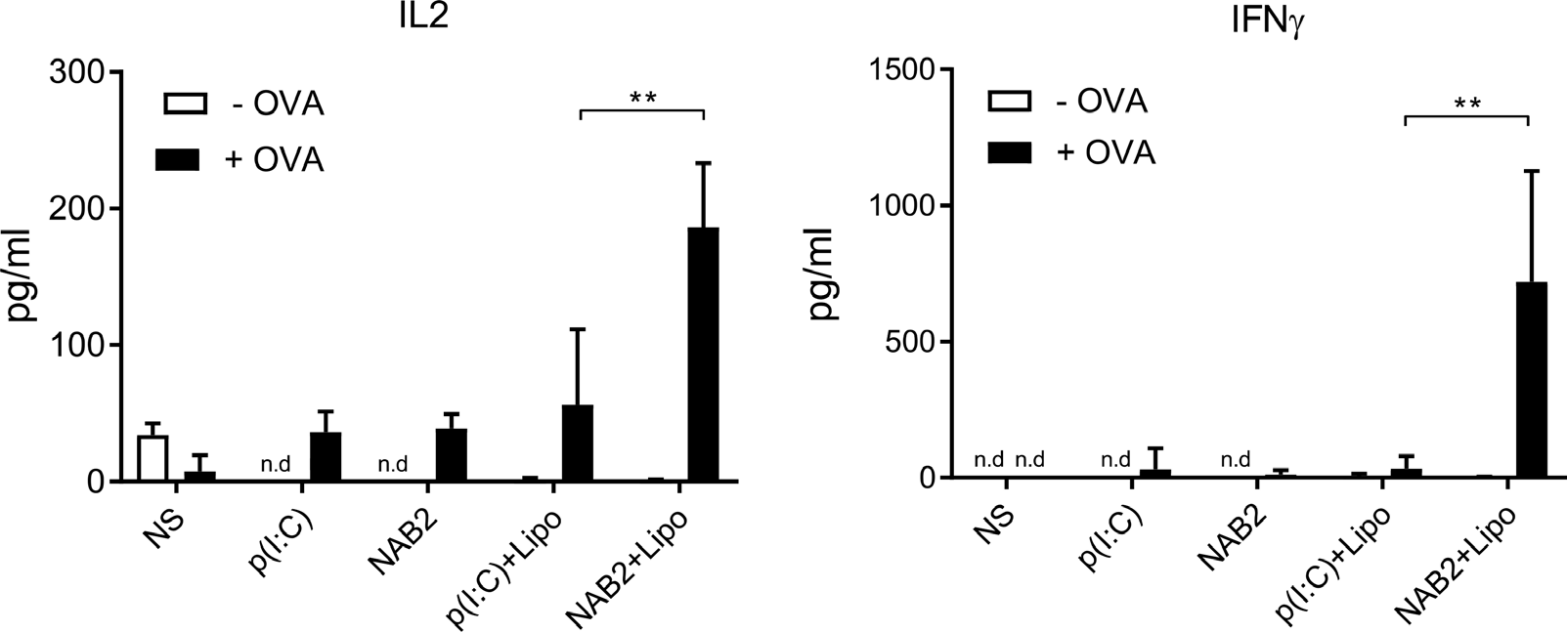
NAB2-stimulated BMDC activate CD8^+^ OT-1 T cells to produce IL2 and IFNγ Levels of IL2 and IFNγ in supernatant of CD8^+^ OT-1 T cells after 3 days of co-culture with OVA peptide-loaded or unloaded BMDC stimulated for 24 h with 2 µg/ml of NAB2 or poly(I:C), with and without Lipofectamine (Lipo), or unstimulated (NS). Cytokines were quantified by ELISA. Plots show cumulative data of two independent experiments. Mean ± SEM of triplicates are shown. Statistical analysis was performed with Student *t*-test. ^**^p < 0.01, n.d., not determined, cytokine levels below detection limit.

To determine the capacity of NAB2-stimulated cells to induce an antigen-specific T-cell response, and considering the increased MHC-I expression upon NAB2 stimulation, we isolated CD8^+^ T cells from OT-I mice and co-cultured them with OVA-pulsed BMDC, which were pre-stimulated with NAB2 or poly(I:C). 3 days later, we measured the amount of IL-2 and IFN-γ in the co-culture supernatant. BMDC that were incubated with NAB2 and lipofectamine induced the highest cytokine production, also in comparison to poly(I:C)-stimulated cells (Figure 2). Of note, in the absence of lipofectamine, NAB2 induced only a marginal IL-2 and IFN-γ production, similarly to poly(I:C)-stimulated BMDC. In the absence of the antigen, we did not detect cytokine production in any of the conditions tested, showing the specificity of the T-cell response. Thus, NAB2-stimulated dendritic cells are able to activate T cells in an antigen-specific manner, and to a greater extent than poly(I:C).

### NAB2 increases IRF3 activation compared to poly(I:C)

**Figure 3:**
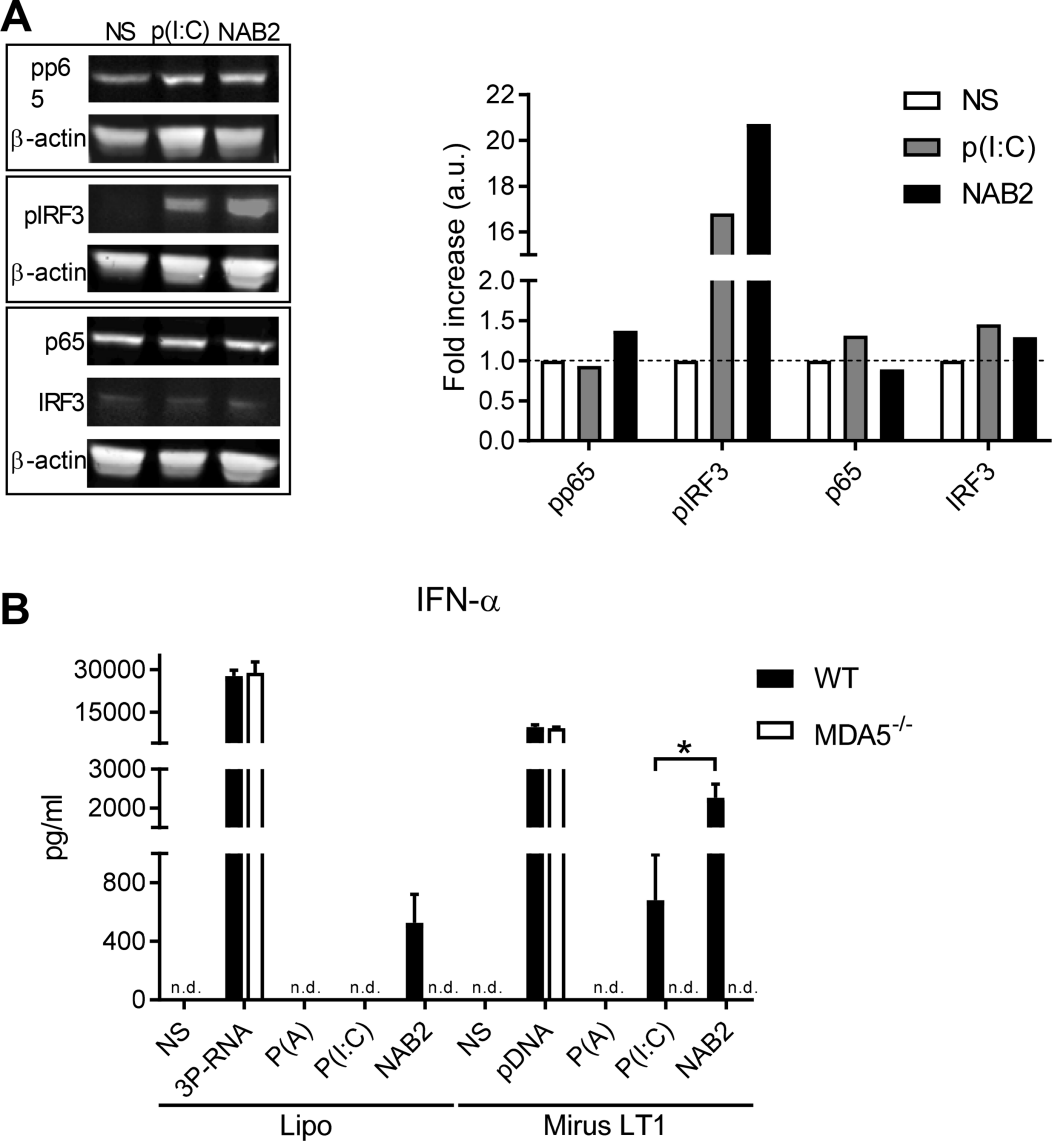
NAB2 increases IRF3 but not NF-κB signalling in murine BMDC *(***A**) Immunoblot analysis of p65 and IRF3 phosphorylation in lysate of BMDC. Cells were stimulated for 2 h with poly(I:C) or NAB2, both complexed with lipofectamine, or not stimulated (NS). Individual blots are depicted by rectangles. Signal intensity is expressed as fold increase referred to unstimulated samples, normalized to β-actin. Blots are representative of two independent experiments. (**B**) IFNα levels in the supernatant of bone marrow cells from WT and MDA-/- mice stimulated for 18 h with NAB2 or poly(I:C) using Lipofectamine 2000^®^ (Lipo) or Mirus TransIT LT1^®^ (Mirus LT1) as transfection reagent. Mean ± SEM of triplicates are shown. Poly (A) was used as negative control. 3P-RNA and DNA were used as positive controls. All the stimulants were used to a final concentration of 200 ng/well. Statistical analysis was performed with Student *t*-test. ^*^p < 0.05, n.d., not determined, cytokine levels below detection limit.

To investigate the mechanism causing the enhanced IFN-α response upon NAB2 stimulation, we performed immunoblot analysis on the cell lysate of murine BMDC stimulated for 2 h with NAB2 or poly(I:C). As both agonists were reported to signal through TLR3 and MDA5 pathways [7, 8, 16], we analysed the activation of the main transcription factors downstream to these receptors. The two agonists induced an equal phosphorylation of the p65 subunit of the transcription factor NF-κB, which is mainly responsible for the expression of pro-inflammatory cytokines, such as IL-6, following TLRs stimulation [19]. In contrast, NAB2 increased the phosphorylation of the transcription factor IRF3, which regulates the expression of type I IFNs following RLR and TLR3 stimulation [20] (Figure 3A). NAB2 and poly(I:C) did not affect the total p65 or IRF3 amount. MDA-5 was below the detection limit in all conditions (data not shown). Thus, the phosphorylation patterns of IRF3 and p65 correlate with the cytokine response observed following NAB2 and poly(I:C) stimulation. Bone marrow cells from mice deficient for MDA-5 produced no IFN-α following NAB2 stimulation, demonstrating that the induction of this cytokine is entirely MDA-5 dependent (Figure 3B). Thus, NAB2 promotes an enhanced type I IFN response in BMDCs by modulating the MDA-5 downstream signalling pathway, and in particular by inducing a stronger IRF3 activation.

### NAB2 increases type I IFN responses *in vivo*

**Figure 4:**
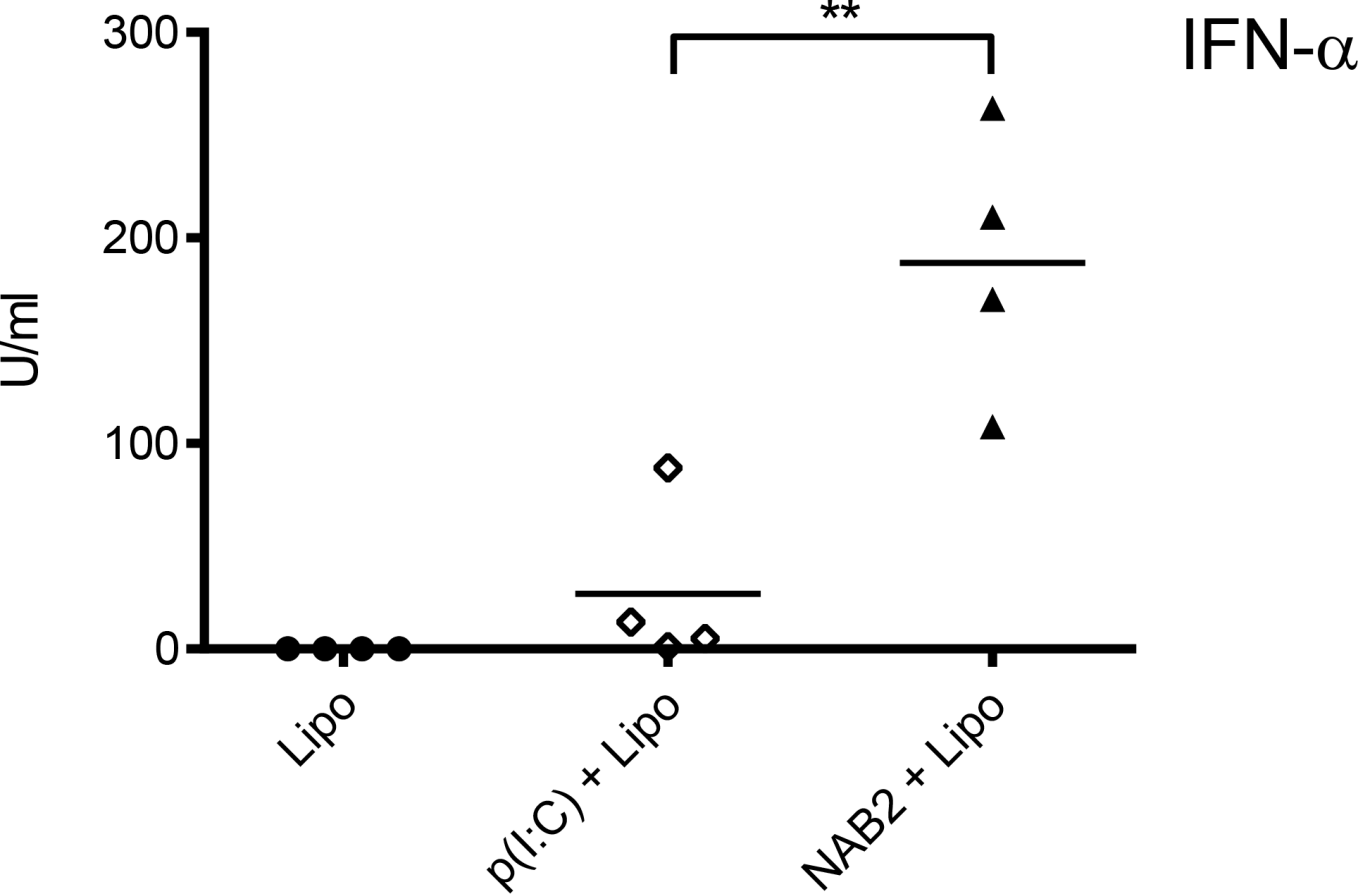
NAB2 increases IFN-α serum levels *in vivo* IFN-α levels in the serum of mice treated with the NAB2 or poly(I:C). Mice were injected i.p. with 50 µg/mouse of poly(I:C) or NAB2 plus Lipofectamine. Cytokines in the serum were measured by ELISA 4 h after the injection. Each dot represents one mouse (*n* = 4). One of two independents experiments is shown. Significance was measured by Student *t*-test. ^**^p < 0.01.

To examine whether the differential response induced by NAB2 and poly(I:C) was detectable *in vivo*, we injected mice with the two PRR agonists, and 4 h later we measured the cytokine levels in the serum. Again, while we did not detect a statistical significant difference in IL-6 production (data not shown), NAB2 induced a markedly increased IFN-α serum expression, compared to poly(I:C) (Figure 4). Of note, we did not observe any signs of toxicity following NAB2 injections. Thus, NAB2 is more efficient than poly(I:C) in promoting a type I IFN response, *in vitro* as well as *in vivo*.

### NAB2 augments the IFN I response of human monocyte-derived macrophages compared to poly(I:C)

**Figure 5:**
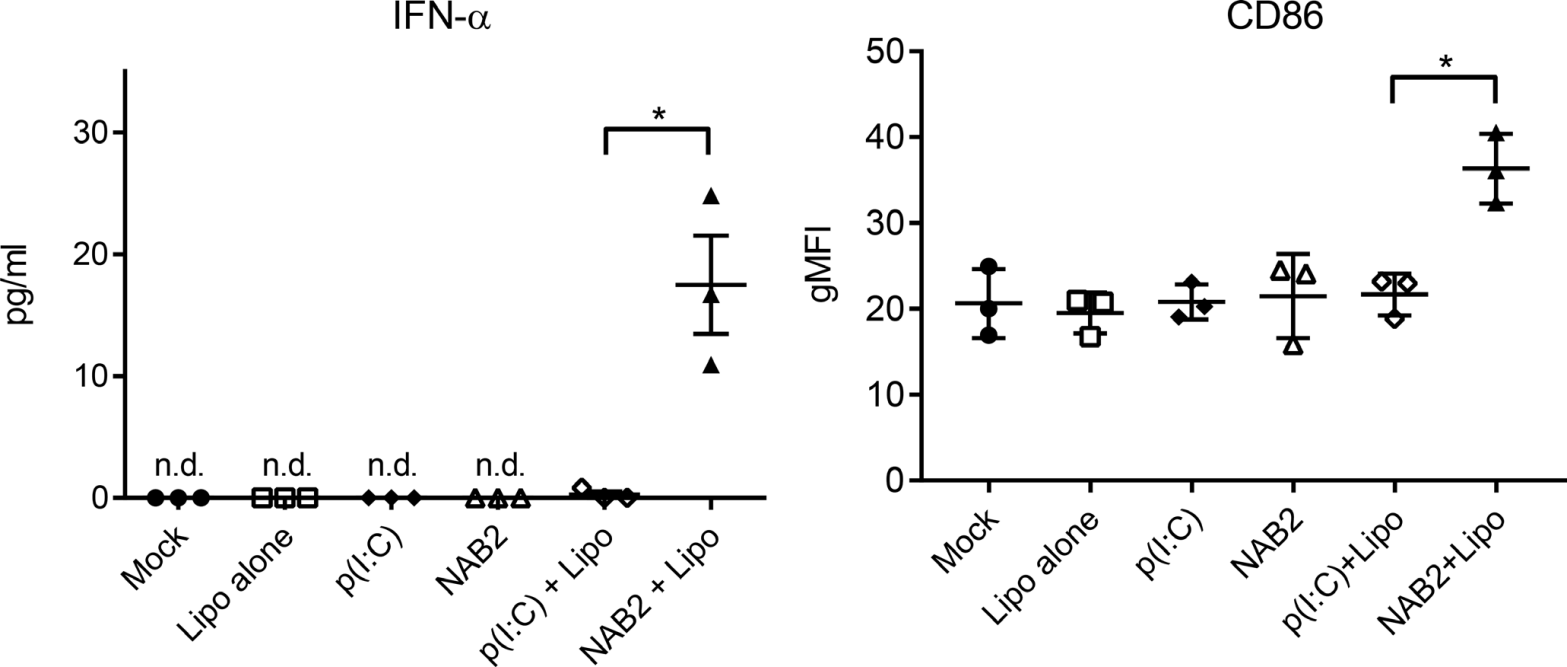
NAB2 enhances IFN-α production and activation of human monocyte-derived macrophages IFN- α levels in the supernatant of human monocyte-derived macrophages (left) and CD86 surface expression (right) on macrophages treated with the indicated agonists for 16 h. IFN-α was measured by Luminex analysis and the CD86 surface expression was measured by flow cytometry. CD86 expression is expressed as geometric mean fluorescence intensity (gMFI). Each dot represents one independent donor (*n* = 3). For each donor, the mean of two independent experiments is shown. Significance was measured by Student *t*-test. ^*^p < 0.05.

To assess whether our observations in the mouse model were valid also in human cells, we stimulated with the two PRRs agonists human monocyte-derived macrophages generated according to Mia et al. [21]. We tested the effect of NAB2 on macrophages because in our previous published [22] and unpublished work, we observed that the anti-tumoral effect of PRR stimulation combined with a prophylactic vaccine was dependent on the activation status and cytokine profile of tumor-infiltrating macrophages. A combination of M-CSF, IL-4/IL-10/TGF-β yielded a phenotype of adherent CD206+CD163+ macrophages with downregulated CD86 expression. We analysed the cell activation state and IFN-α production following stimulation. Similarly to the mouse system, the stimulation with NAB2 induced a marked increase of IFN-α production (Figure 5, left) and CD86 expression on human macrophages (Figure 5, right). In contrast, poly(I:C) did not cause any detectable effect. We did not detect IFN-α production or CD86 up-regulation upon stimulation in the absence of the transfection reagent. Taken together, our observations suggest that NAB2 induces a consistent and more potent type I IFN response compared to poly(I:C) both in human cells and in mice.

## DISCUSSION

Vaccine formulations that exploit the nucleic acid-sensing mechanisms of immune cells can be used to maximize the response against cancer and chronic infections, mainly for their capability to induce a potent type I IFN response. Indeed, type I IFNs are crucial mediators of the anti-tumoral immune response, promoting DC maturation, antigen cross-presentation and in turn activation of cytotoxic T lymphocytes, NK cells activation and polarization of Th1 cells [23]. Moreover, the ability of type I IFNs to prime spontaneous anti-tumoral T-cell responses [24] makes these pleiotropic cytokines, and the mechanisms that drive their production highly attractive targets of cancer immunotherapeutic approaches [25].

The synthetic dsRNA poly(I:C) is currently considered the most potent inducer of type I IFNs among all TLRs agonists [26]. However, poly(I:C) limitations, such as batch-dependent variability and high toxicity, hampered its use in clinical settings and highlight the need for new and more efficient TLR3 and MDA-5 targeting adjuvants. In this work we show that NAB2, a yeast-derived dsRNA molecule that also signals through TLR3 and MDA-5, is able to induce a stronger IFN-α production and innate immune cell activation compared to poly(I:C), both in the mouse and human systems. Moreover, since the production process of NAB2 is based on the isolation of viral genomic RNA, the length of the molecule is constant in different batches, assuring highly reproducible results (Supplementery Figure 4). NAB2 also induces a higher CXCL10 production, in spite of a similar IL-6 expression. While the comparable IL-6 expression may be due to the similar capacity of NAB2 and poly(I:C) to activate the NF-κB pathway, the different CXCL10 production suggests that its expression is driven by NAB2 mainly through IRF3 activation. However, it is also possible that CXCL10 production is induced in a paracrine manner by type I IFNs [27]. Considering a) that CXCL10 expression is associated with Th1 immune responses and b) the role of CXCL10 in attracting activated T cells [28, 29], NAB2 appears as a promising adjuvant to promote cell-mediated immunity. Indeed, we additionally demonstrate the potentiated antigen-presenting function of NAB2-stimulated BMDC *in vitro*: MHC-I surface expression increases upon NAB2 stimulation, and BMDC gain the capacity to activate CD8^+^ T cells in an antigen-specific manner to produce IL-2 and IFN-γ. Our data indicate that NAB2 represents a novel immune stimulant with a high therapeutic potential, especially in the context of cancer immunotherapy, although further studies are needed to test the adjuvant potential of NAB2 *in vivo*.

Of note, we observed that NAB2 promoted a stronger IFN-α expression compared to poly(I:C) only when the two agonists were complexed with lipofectamine, a transfection reagent which allows their direct entry in the cytoplasm. On the contrary, the two PRRs induced a similar and only marginal amount of IFN-α when applied without carrier. It was previously shown that, when poly(I:C) and NAB2 are complexed with lipofectamine, the main receptor that is triggered is the RLR MDA-5 [16, 17]. We could confirm this here using immune cells from MDA5-deficient mice. In line with this, delivery of NAB2 to the cytoplasm by using the alternative carrier TransIT^®^-LT2 (Mirus) produced similar results in terms of IFN-α production by human PBMCs (Supplementery Figure 3). DOTAP, which delivers nucleic acids to the endosome, was less effective. Altogether these observations suggest that NAB2 is a more efficient MDA-5 agonist than poly(I:C), while they induce a similar TLR3-dependent response. In line with this, NAB2 might exert a different function compared to poly(I:C) depending on the cell type, as a consequence of different cellular distribution of the MDA-5 and TLR3 receptors [9]. For example, compared to poly(I:C), NAB2 might have a major impact on NK cells activation, since this cell type crucially relies on MDA-5 for nucleic acid sensing, whereas TLR3 plays only a marginal function [30].

The mechanism by which NAB2-stimulation improves the MDA-5-dependent response remains to be defined. Basal expression of MDA-5 is usually low, and it can be induced by cytokines such as IFN-β and TNF-α [31]. However, it is unlikely that the increased MDA-5 signalling induced by NAB2 is due to an up-regulation of the receptor expression, since we could not detect a significant MDA-5 expression in any of the conditions tested, even upon stimulation by NAB2 or poly(I:C). IRF3 is the major transcription factor that regulates type I IFN responses upon nucleic acid sensing in innate immune cells [19]. We observed an increased IRF3 phosphorylation upon NAB2 stimulation, but a similar NF-κB activation induced by the two agonists. NAB2 might thus impact the MDA-5 downstream signalling at the level where the pathways involving IRFs or NF-κB diverge. Upon dsRNA recognition, MDA-5 interacts with IPS-1, or Mitochondrial antiviral-signaling adaptor protein (MAVS) [32], which is considered the point of divergence of the two pathways. Following interaction with MDA-5, IPS-1 may activate the TBK1/IKKε kinases, which in turn phosphorylate IRF3 [33], or alternatively activate IKK, and finally NF-κB, in a signalling cascade involving FADD-RIP1 and caspase-8/10 [34]. Further experiments are needed to fully elucidate the signalling events leading to IRF3 phosphorylation.

On the structural level, MDA-5 recognizes long dsRNA molecules by cooperatively assembling into helical filaments [35]. The length and lifetime of MDA-5 filaments is determined also by the length of the dsRNA molecule, and this is a crucial factor in determining the MDA-5 signalling output [36]. Thus, a possible explanation of the improved MDA-5-dependent response upon NAB2 stimulation is the increased length of the NAB2 molecule, compared to poly(I:C).

Different-size poly(I:C)s are commercially available, usually referred to as high (HMW) or low molecular weight (LMW) poly(I:C)s, with a respective average size of 1.5–8 kb and 0.2–1 kb. Both poly(I:C) formats are able to signal through TLR3 and MDA-5, but there are contrasting results concerning their relative efficacy [37, 38]. In this work we compare NAB2 with LMW poly(I:C), because, from previous observations of our group, LMW poly(I:C) produces more reproducible results compared to HMW poly(I:C), with regard to its innate immune cell-stimulant activity ([17] and unpublished results). Moreover, we previously showed how LMW poly(I:C) has a strong anti-tumoral effect in an active immunotherapy approach [39]; taken together, we thus consider LMW poly(I:C) a proper and effective control for testing NAB2 immune stimulant activity in pre-clinical experiments. However, it would be of interest to compare NAB2 immune stimulatory activity with other poly(I:C) formulations.

In conclusion, considering the strong type I IFN response induced, NAB2 has the potential to be a more efficient adjuvant than poly(I:C) for therapeutic immunization against cancer or infections. Our work provides the rationale to compare the efficiency of NAB2 and poly(I:C) in a therapeutic setting, such as the anti-tumoral effect of their administration in mouse tumor models. However, it is important to keep in mind that the most prominent adverse effect of the use of poly(I:C) or poly(I:C)-derived compounds in clinical settings are attributed to the induction of systemic inflammatory responses. Although we observed no signs of toxicity in mice receiving high doses of NAB2, further studies are needed to carefully evaluate the potential side effects of NAB2-mediated immune stimulation, and to test the use of more targeted delivery strategies [40, 41].

## MATERIALS AND METHODS

### Human monocyte purification and immune stimulation

Human monocytes were isolated and purified from peripheral blood mononuclear cells (PBMCs) of buffy coats obtained from healthy donors (Etablissement Français du Sang, Strasbourg) using Ficoll-Paque PLUS (GE Healthcare) according to the manufacturer’s instructions. After isolation from PBMCs either by positive CD14+ selection or by negative selection using monocyte isolation kit II (Miltenyi, Biotec), 4 × 10^5^ monocytes were cultured on 48-well plates in 500 µl Macrophage Base Medium DXF (Promocell) supplemented with 50 ng/ml M-CSF (Miltenyi Biotec). Medium was changed at day 3 and day 7. At day 7, CD16^hi^CD68^+^CD11b^+^ M0 macrophages were polarized towards a M2 phenotype adding IL-4, IL-10 and TGF-beta (Miltenyi Biotec) to a concentration of 20 ng/ml. Two days later, CD163^+^ CD206^+^ M2 macrophages were treated with NAB2 or poly(I:C) (LMW (tlrl-picw, In*vivo*gen)) mixed or not with Lipofectin reagent (Invitrogen) in Opti MEM medium (Gibco, Thermo Fisher). 50 µl of the respective mix were added per well for 16 h. For testing different transfection reagents, freshly isolated PBMCs were stimulated with NAB2 transfected with Lipofectamine (Invitrogen, Thermo Fisher Scientific), DOTAP (Carl Roth) and TransIT^®^-LT2 (Mirus Bio LLT) diluted in Opti MEM (Gibco, Thermo Fisher Scientific). Supernatants were collected after 24 h for cytokine detection.

### Mice and treatments

Wild-type C57BL/6J mice were purchased from Janvier labs (Le Genest-St-Isle, France). MDA-5-deficient mice (C57BL/6 background) and WT controls were from the University of Bonn. Mice were maintained in specific-pathogen free conditions and used between 6-14 weeks of age. In the indicated experiments, mice were injected i.p. with 50 µg/mouse of poly(I:C) low molecular weight (In*vivo*gen, Toulouse, France), or 50 µg/mouse NAB2 (Transgene S.A., Illkirch-Graffenstaden, France), complexed with Lipofectamine RNAiMAX (Invitrogen, Carlsbad, CA). 4 h after the injection, mice were euthanized, and blood was collected by cardiac puncture, for the analysis of serum cytokines. All animal experimentation procedures were performed according to the Swiss or German federal legislation.

### Murine cell culture and immune stimulation

Primary cells from bone marrow and spleen were obtained as described in [42]. To prepare conventional BMDCs, bone marrow cells were cultured in RPMI 1640 medium, 50 U/mL penicillin, 50 µg/mL streptomycin, 2 mmol/l L-glutamine (all from PAA laboratories, Pasching, Austria), 10% (v/v) fetal calf serum (Life Technologies, Grand Island, NY), supplemented with 20 ng/ml GM-CSF (PeproTech, Rocky Hill, NJ). On day 6, loosely adherent cells and cells in suspension were harvested and used for the indicated experiments. For cell stimulations, a total of 2 × 10^5^ BMDCs or 2–4 × 10^5^ primary bone marrow cells and splenocytes per well were seeded in 96-well plates. Stimulation of the cells was performed in complete medium, with the agonists at the concentrations indicated in the figure legends for 18-24 h. The same 2 h stimulation protocol was performed for flow cytometry analysis, cytokine production, and for the analysis of the activation of intracellular signalling pathways. Low molecular weight poly(I:C) and R848 were purchased respectively from Invi vogen and Enzo Life Sciences (New York, NY). NAB2 was provided by Transgene S.A. Poly(I:C), NAB2, 3P-RNA and pDNA transfections were performed with Lipofectamine (Life Technologies), TransIT-LT1 or LT2 (Mirus Bio LLT) according to manufacturers’ specifications.

### Flow cytometry and ELISA

For flow cytometry analysis, cells were incubated for 10 min with Fc receptor-blocking antibody (TruStain fcX, Biolegend, San Diego, CA) and stained with fluorescently labeled antibodies. Anti-mouse CD11b (M1/70), CD11c (N418), MHC-II (M5/114.15.2), CD80 (16-10A1) and CD86 (GL-1) were used. Pacific blue-coupled Armenian hamster IgG antibody (HTK888), phycoerythrin-coupled rat IgG2a,κ (RTK2758) and APC-Cy7-coupled rat IgG2b,κ (RTK4530), were used as isotype controls. All these antibodies were purchased from Biolegend (San Diego, CA). Anti-mouse CD40 (1C10), PDL1 (MIH5), MHCI (AF6-885.5.3) were purchased from eBioscience (Thermo Fisher Scientific). All cell acquisitions were recorded using the MACSQuant system from Miltenyi Biotec (Bergisch Gladbach, Germany) or the NovoCyte Flow Cytometer (ACEA Biosciences). Data were analyzed using FlowJo vX.0.7 software (Tree Stat, Inc., Ashland, USA). For the flow cytometry analysis of human monocytes, cells were incubated for 20 min with human FcR Blocking Reagent (Miltenyi Biotec) and APC-labeled anti-CD86 (clone FM95, Miltenyi Biotec). Dead cells were labeled by automatic propidium iodide staining before cell acquisition recorded using MACSQuant cytometer (Miltenyi). Data were analyzed using Kaluza software (Beckman Coulter). Cytokine levels in the supernatant of stimulated cells and serum of treated mice were quantified by sandwich ELISA, following the kit protocols (BD Biosciences and BioLegend, San Diego, CA). For human monocytes, the supernatant of stimulated cells was taken and IFN-alpha levels were measured (Human IFN-alpha Simplex, ProcartaPlex, eBiosciences) on a MAGPIX device (Luminex XMAP Technologies).

### Immunoblot analysis

BMDCs (4 × 10^6^/well) were seeded in 24-well plates and stimulated in serum-free Opti-MEM (Life Technologies, Invitrogen). Immunoblot analysis was then performed as described in [17]. The following primary antibodies were used: anti–β-actin (8H10D10), anti–phospho-p65 (Ser536, 93H1), anti-p65 (L8F6 or D14E12), anti–phospho-IRF3 (4D4G), anti-IRF3 (D83B9), and anti–MDA-5 (D74E4). Membranes were analyzed in a Li-Cor fluorescence reader (Li-Cor, Lincoln, NE).

### Stimulation of OT1 CD8+ T cells

CD8+ T cells were purified from spleens of OT I mice (C57BL/6-Tg(TcraTcrb)1100Mjb/Crl, Charles River Labs) by negative selection with the CD8a+ mouse T cell isolation kit (Miltenyi Biotec). Separation efficiency was evaluated by FACS. Purified CD8+ T cells (1 × 10^5^ cells/well) were co-cultured with BMDCs (5 × 10^4^/well) stimulated as described above, in presence or absence of OVA peptide (25 µg/ml). Supernatants were collected after three days of co-culture for cytokine detection.

### Statistical analysis

Statistical significance was evaluated using two-tailed Student’s *t* test, comparing the mean ± SEM of at least 3 biological replicates per conditions. *P*-values < 0.05 were considered significant. Each experiment was repeated at least twice. Statistical analysis was done with GraphPad Prism 5 software (GraphPad Softwares Inc., CA).

## SUPPLEMENTARY MATERIALS FIGURES


