# Supplementary material for: NAB2 is a novel immune stimulator of MDA-5 that promotes a strong type I interferon response
Source: Oncotarget. 2017 Dec 15;9(5):5641–51. doi: 10.18632/oncotarget.23725 (PMC5814164; doi:10.18632/oncotarget.23725)
Supplement: Supplementary file 1 [file oncotarget-09-5641-s001.pdf]

NAB2 is a novel immune stimulator of MDA-5 that promotes a strong type I IFN response

SUPPLEMENTARY MATERIALS

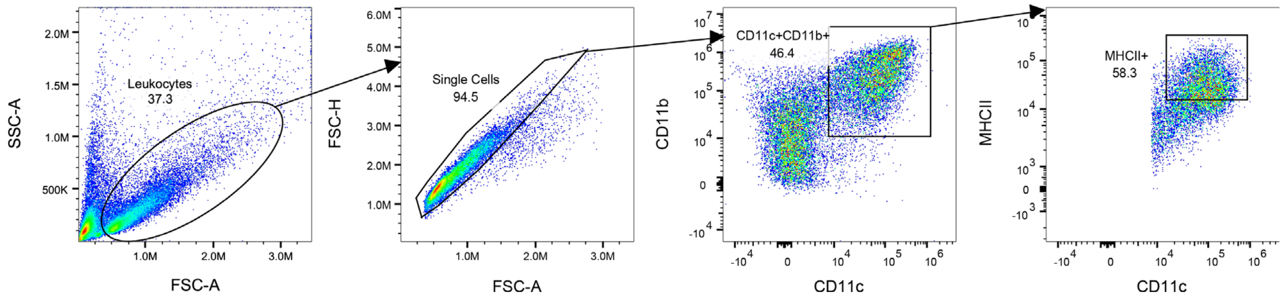

Supplementary Figure 1: Mouse dendritic cell gating strategy. Plots show the sequential gating strategy used to analyse the expression of activation markers on mouse DC. Leukocytes are gated by means of FSC-A and SSC-A values, doublets are excluded according to FSC-A and FSC-H values, CD11b+ and CD11c+ are then selected and DC are defined as MHCII+CD11b+CD11c+ cells. The same gating strategy is used to analyse BMDC and splenic DC.

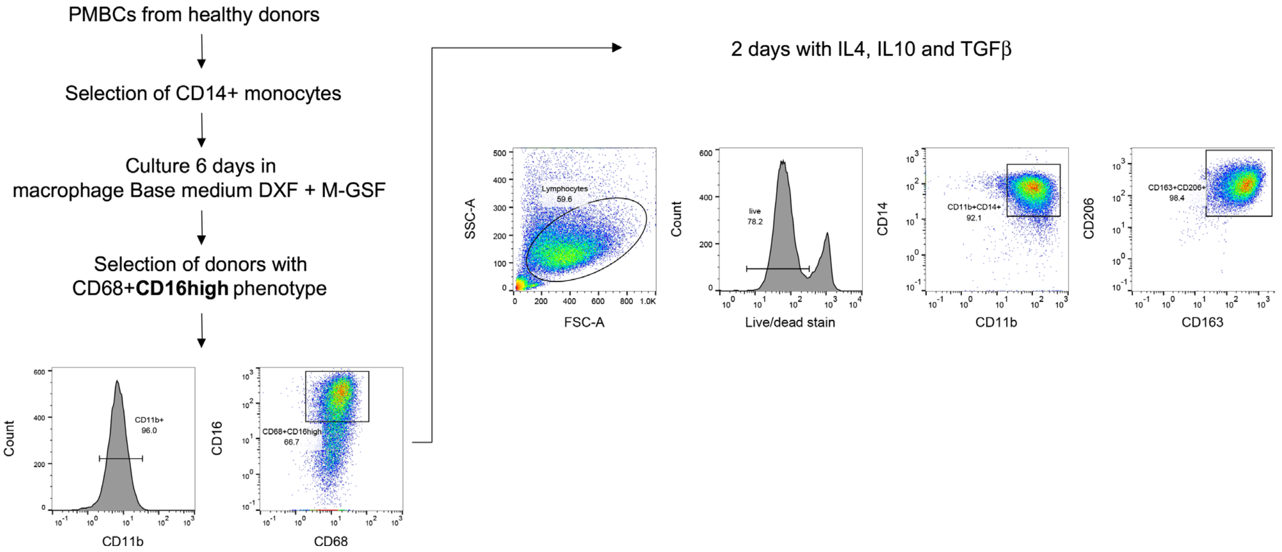

Supplementary Figure 2: Human monocyte-derived macrophages gating strategy. Summary of isolation of human monocytes and analysis of monocyte-derived M2-macrophages. Human CD14+ monocytes are separated from PBMCs isolated from healthy donors and cultured with a macrophage-differentiation inducing medium. CD11b+CD68+CD16high cells are further cultured with IL-4, IL-10 and TGFb. Finally macrophages are selected within the leukocyte gate defined on FSC-A and SSC-A values. PI-negative live cells are selected and macrophages are defined as CD11b+CD14+ cells. CD206+CD163+ macrophages are finally gated for the analysis of the expression of surface activation markers.

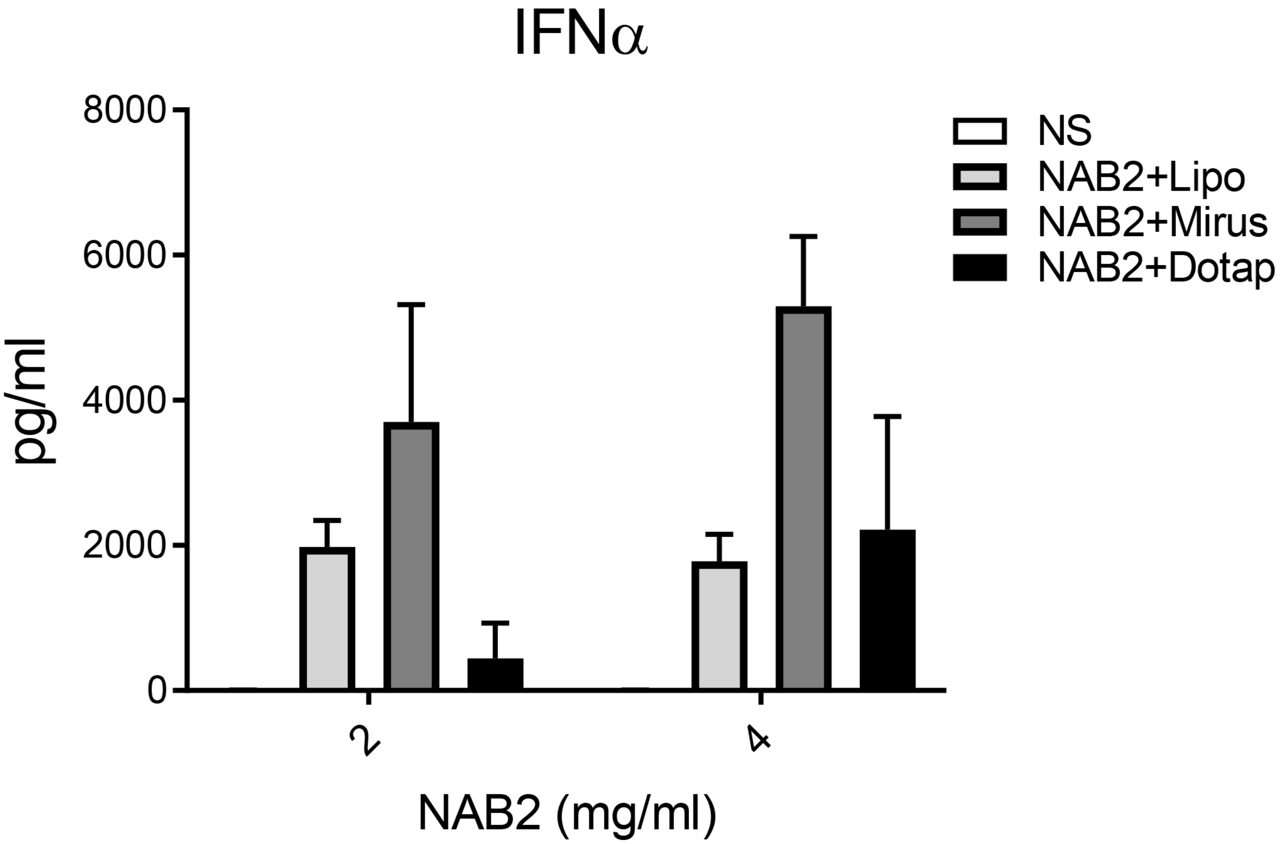

Supplementary Figure 3: NAB2-stimulated PBMCs transfected with different delivery systems produces comparable amounts of INFα. Production of human IFNα detected in supernatants of freshly isolated PBMCs stimulated with (2 µg/ml or 4 µg/ml) of NAB2 in combination with Lipofectamine (Lipo), TransIT®-LT2 (Mirus) or Dotap for 24h. Plots represent pooled data from two healthy donors.

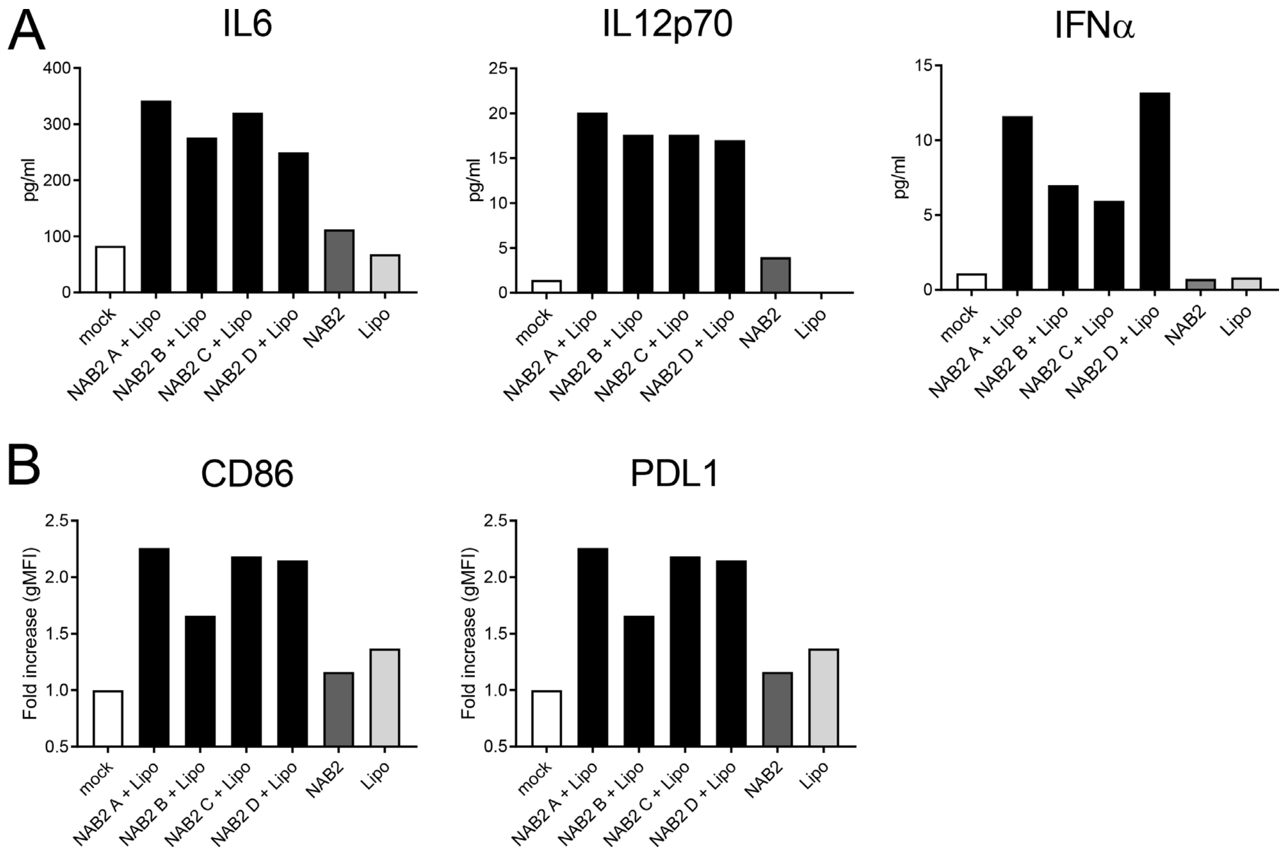

Supplementary Figure 4: Stimulation of human monocyte-derived macrophages with NAB2 shows little batch-dependent variation. (**A**) Production of IL6, IL12p70 and IFNα in supernatants of human monocyte-derived macrophages stimulated with four different batches of NAB2 (NAB2 A-D) with Lipofectamine (Lipo), NAB2 B without Lipofectamine or Lipofectamine alone. Plots show representative data of one out of two healthy donors. (**B**) Expression of the markers CD86 and PDL1 of human monocyte-derived macrophages stimulated as indicated above and measured by flow cytometry. The plots represent data from one out of four healthy donors expressed as fold change increase of the geometric mean fluorescence intensity (gMFI) compared to control (mock).
